# Supplementary material for: Integrins Have Cell-Type-Specific Roles in the Development of Motor Neuron Connectivity
Source: J Dev Biol. 2019 Aug 27;7(3):17. doi: 10.3390/jdb7030017 (PMC6787651; doi:10.3390/jdb7030017)
Supplement: Supplementary file 1 [file jdb-07-00017-s001.pdf]

Journal of Developmental Biology

MDPI

Supplemental Table 1. *C. elegans* strains used in this study

Strain	Genotype	Description	Plasmid
IZ2489	*ufIs34; vsIs48*	GABA-specific (including DD + VD) transcriptional reporter (red); Cholinergic-specific transcriptional reporter (green)	P*unc-47*::mCherry *(pPRB5), lin-15+(pL15Ek);* P*unc-17*::GFP, *lin-15*+(pL15Ek)
IZ1481	*ina-1(gm144)III;ufIs34;vsIs48*	hypomorphic *ina-1* allele; GABA-specific (including DD + VD) transcriptional reporter (red), cholinergic-specific transcriptional reporter (green)	P*unc-47*::mCherry *(pPRB5), lin-15+(pL15Ek);* P*unc-17*::GFP, *lin-15*+(pL15Ek)
IZ1310	*ufEx432*	DVA specific transcriptional reporter	P*nlp-12*::mCherry (pCL27)
IZ1430	*ina-1(gm144); ufEx432*	hypomorphic *ina-1* allele; DVA specific transcriptional reporter	P*nlp-12*::mCherry (pCL27)
IZ2288	*oxIs12; ufIs128*	DD-specific transcriptional reporter (red); GABA-specific (including DD + VD) transcriptional reporter (green)	P*unc-47*::GFP; P*flp-13*::mCherry (pAP31)
IZ3727	*ina-1(gm144)III;oxIs12;ufIs128*	*ina-1* hypomorphic allele; DD-specific transcriptional reporter (red); GABA-specific (including DD + VD) transcriptional reporter (green)	P*unc-47*::GFP; *Pflp-13*::mCherry (pAP31)
IZ3786	*ina-1(gm144)III;oxIs12;ufIs128; ufEx1582*	GABA-specific (including DD + VD) transcriptional reporter (green); DD-specific transcriptional reporter (red); endogenous *ina-1* promoter driving *ina-1*+ rescue in *ina-1(gm144)* background	*Punc-47*::GFP; *Pflp-13*::mCherry (pAP31); *Pina-1*::*ina-1cDNA* (pDO91)
IZ3787	*ina-1(gm144)III;oxIs12;ufIs128; ufEx1583*	GABA-specific (including DD + VD) transcriptional reporter (green); DD-specific transcriptional reporter (red); endogenous *ina-1* promoter driving *ina-1*+ rescue in *ina-1(gm144)* background	P*unc-47*::GFP; P*flp-13*::mCherry (pAP31); P*ina-1*::*ina-1cDNA* (pDO91)

IZ3790	*ina-1(gm144)III;oxIs12;ufIs128; ufEx1586*	GABA-specific (including DD + VD) transcriptional reporter (green); DD-specific transcriptional reporter (red); pan-neuronal promoter driving *ina-1*+ rescue in *ina-1(gm144)* background	P*unc-47*::GFP; P*flp-13*::mCherry (pAP31); P*rgef-1::ina-1cDNA* (pDO81)
IZ3778	*ina-1(gm144)III;oxIs12;ufIs128; ufEx1577*	GABA-specific (including DD + VD) transcriptional reporter (green); DD-specific transcriptional reporter (red); pan-neuronal promoter driving *ina-1*+ rescue in *ina-1(gm144)* background	P*unc-47*::GFP; P*flp-13*::mCherry (pAP31); P*rgef-1::ina-1cDNA* (pDO81)
IZ3833	*ina-1(gm144)III;oxIs12;ufIs128; ufEx1611*	GABA-specific (including DD + VD) transcriptional reporter (green); DD-specific transcriptional reporter (red); pan-neuronal promoter driving *ina-1*+ rescue in *ina-1(gm144)* background	P*unc-47*::GFP; P*flp-13*::mCherry (pAP31); P*rgef-1::ina-1cDNA* (pDO81)
IZ3817	*ina-1(gm144)III;oxIs12;ufIs128; ufEx1604*	GABA-specific (including DD + VD) transcriptional reporter (green); DD-specific transcriptional reporter (red); GABAergic promoter driving *ina-1*+ rescue in *ina-1(gm144)* background	P*unc-47*::GFP; P*flp-13*::mCherry (pAP31); P*unc-47::ina-1cDNA* (pDO117)
IZ3814	*ina-1(gm144)III;oxIs12;ufIs128; ufEx1601*	GABA-specific (including DD + VD) transcriptional reporter (green); DD-specific transcriptional reporter (red); GABAergic promoter driving *ina-1*+ rescue in *ina-1(gm144)* background	P*unc-47*::GFP; P*flp-13*::mCherry (pAP31); P*unc-47::ina-1cDNA* (pDO117)
IZ3831	*ina-1(gm144)III;oxIs12;ufIs128; ufEx1610*	GABA-specific (including DD + VD) transcriptional reporter (green); DD-specific transcriptional reporter (red); GABAergic promoter driving *ina-1*+ rescue in *ina-1(gm144)* background	P*unc-47*::GFP; P*flp-13*::mCherry (pAP31); P*unc-47::ina-1cDNA* (pDO117)
IZ3832	*ina-1(gm144)III;oxIs12;ufIs128; ufEx1611*	GABA-specific (including DD + VD) transcriptional reporter (green); DD-specific transcriptional reporter (red); GABAergic promoter driving *ina-1*+ rescue in *ina-1(gm144)* background	P*unc-47*::GFP; P*flp-13*::mCherry (pAP31); P*unc-47::ina-1cDNA* (pDO117)
IZ3784	*ina-1(gm144)III;oxIs12;ufIs128; ufEx1580*	GABA-specific (including DD + VD) transcriptional reporter (green); DD-specific transcriptional reporter (red); cholinergic promoter driving *ina-1*+ rescue in *ina-1(gm144)* background	P*unc-47*::GFP; P*flp-13*::mCherry (pAP31); P*unc-17β::ina-1cDNA* (pDO75)

IZ3782	*ina-1(gm144)III;oxIs12;ufIs128; ufEx1579*	GABA-specific (including DD + VD) transcriptional reporter (green); DD-specific transcriptional reporter (red); cholinergic promoter driving *ina-1*+ rescue in *ina-1(gm144)* background	P*unc-47*::GFP; P*flp-13*::mCherry (pAP31); P*unc-17β*::*ina-1cDNA* (pDO75)
IZ3791	*ufEx1587;ufIs34*	Transcriptional *ina-1* reporter (green); GABAergic transcriptional reporter (red)	pDO119 *(*P*ina-1*::GFP) P*unc-47*::mCherry *(pPRB5), lin-15*+(pL15Ek)
IZ3770	*ufEx1574;ufIs43*	Transcriptional *ina-1* reporter (green); cholinergic transcriptional reporter (red)	pDO119*(*P*ina-1*::GFP); P*acr-2*::mCherry (pPRB6)
IZ1876	*gmIs5;ufIs34*	Translational INA-1 reporter (green); GABA-specific (including DD + VD) transcriptional reporter (red)	P*ina-1*::GFP + *rol-6(su1006)*; P*unc-47*::mCherry (pPRB5), *lin-15*+(pL15Ek)
IZ3754	*gmIs5:ufIs43*	Translational INA-1 reporter (green); cholinergic-specific transcriptional reporter (red)	P*ina-1*::GFP + *rol-6(su1006)*; P*acr-2*::mCherry (pPRB6)

Journal of Developmental Biology

MDPI

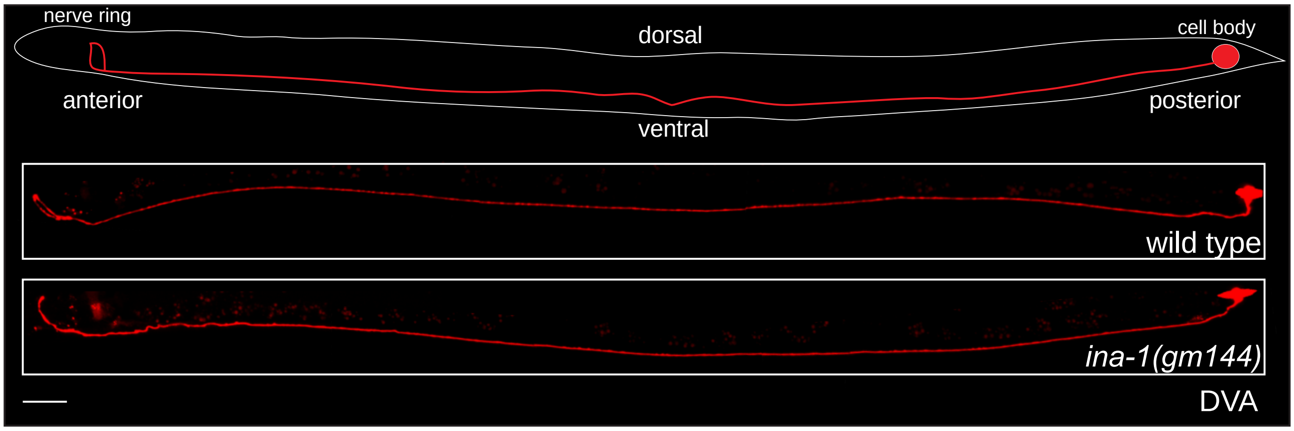

Figure S1. DVA develops independently of *ina-1.*
Top, Schematic of the DVA neuron. *Bottom panels,* Confocal images of DVA axon in wild type (n=28) and *ina-1(gm144)* animals (n=28). Scale bar, 20 µm.

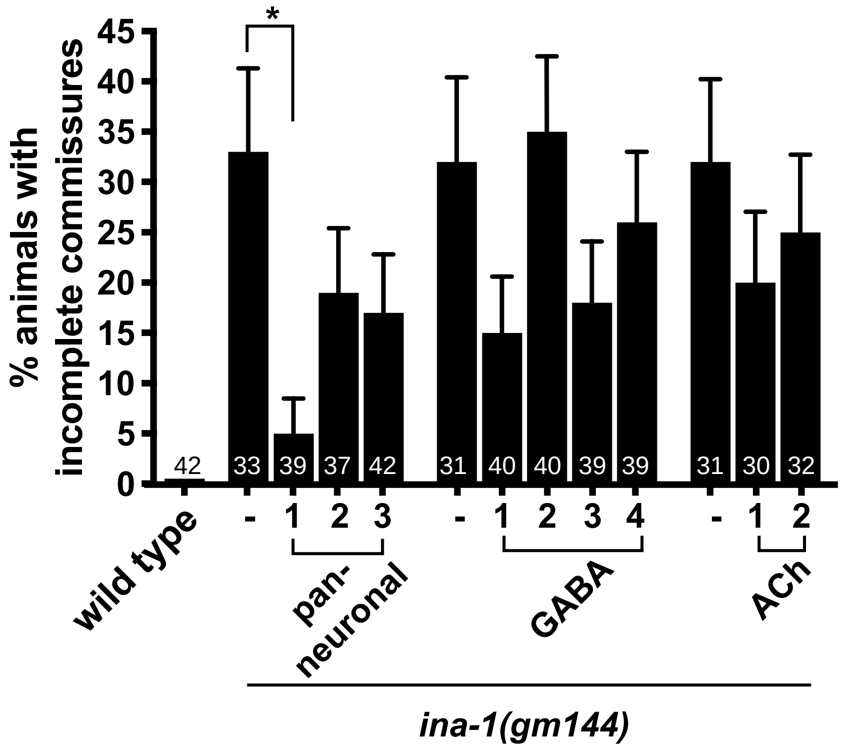

Figure S2. Quantification of incomplete commissures with cell-specific expression of wild type *ina-1* in *ina-1(gm144)* mutants. Promoters for pan-neuronal (P*rgef-1*), GABAergic (P*unc-47*), or cholinergic (P*unc-17β)* were used to drive expression of wild *ina-1* cDNA in *ina-1(gm144)* mutants. Data are plotted as the percentage of incomplete GABAergic commissures for each genotype. Error bars indicate standard error of the proportion. Pan-neuronal expression line #1 provided significant rescue. *p<0.02, Fisher's exact test with Bonferroni correction for multiple comparisons.
